# Pancreaticobiliary Diseases with Severe Complications as a Rare Indication for Emergency Pancreaticoduodenectomy: A Single-Center Experience and Review of the Literature

**DOI:** 10.3390/jcm12175760

**Published:** 2023-09-04

**Authors:** Maximilian Fickenscher, Oleg Vorontsov, Thomas Müller, Boris Radeleff, Christian Graeb

**Affiliations:** 1 Department of General-, Visceral- and Thoracic Surgery, Sana Hospital Hof, 95032 Hof, Germany; 2 Department of Gastroenterology, Sana Hospital Hof, 95032 Hof, Germany; 3 Department of Diagnostic and Interventional Radiology, Sana Hospital Hof, 95032 Hof, Germany

**Keywords:** emergency, pancreaticoduodenectomy, whipple procedure, pancreatic surgery, duodenal ulcer, perforation, acute bleeding, pancreas

## Abstract

The pancreaticobiliary system is a complex and vulnerable anatomic region. Small changes can lead to severe complications. Pancreaticobiliary disorders leading to severe complications include malignancies, pancreatitis, duodenal ulcer, duodenal diverticula, vascular malformations, and iatrogenic or traumatic injuries. Different therapeutic strategies, such as conservative, interventional (e.g., embolization, stent graft applications, or biliary interventions), or surgical therapy, are available in early disease stages. Therapeutic options in patients with severe complications such as duodenal perforation, acute bleeding, or sepsis are limited. If less invasive procedures are exhausted, an emergency pancreaticoduodenectomy (EPD) can be the only option left. The aim of this study was to analyze a single-center experience of EPD performed for benign non-trauma indications and to review the literature concerning EPD. Between January 2015 and January 2022, 11 patients received EPD due to benign non-trauma indications at our institution. Data were analyzed regarding sex, age, indication, operative parameters, length of hospital stay, postoperative morbidity, and mortality. Furthermore, we performed a literature survey using the PubMed database and reviewed reported cases of EPD. Eleven EPD cases due to benign non-trauma indications were analyzed. Indications included peptic duodenal ulcer with penetration into the hepatopancreatic duct and the pancreas, duodenal ulcer with acute uncontrollable bleeding, and penetration into the pancreas, and a massive perforated duodenal diverticulum with peritonitis and sepsis. The mean operative time was 369 min, and the median length of hospital stay was 35.8 days. Postoperative complications occurred in 4 out of 11 patients (36.4%). Total 90-day postoperative mortality was 9.1% (1 patient). We reviewed 17 studies and 22 case reports revealing 269 cases of EPD. Only 20 cases of EPD performed for benign non-trauma indications are reported in the literature. EPD performed for benign non-trauma indications remains a rare event, with only 31 reported cases. The data analysis of all available cases from the literature revealed an increased postoperative mortality rate of 25.8%. If less invasive approaches are exhausted, EPD is still a life-saving procedure with acceptable results. Performed by surgeons with a high level of experience in hepatobiliary and pancreatic surgery, mortality rates below 10% can be achieved.

## 1. Introduction

The pancreaticobiliary system is a complex anatomic region with central tasks in the digestive tract. Digestive enzymes of the exocrine pancreas and bile from the liver drain into the duodenum through the ampulla of Vater. This is essential for the digestion of ingested food [1]. The complex pancreaticobiliary system is vulnerable to organ changes. Small changes can lead to severe complications [2]. Pancreaticobiliary disorders leading to severe complications are malignancies, pancreatitis, duodenal ulcer, duodenal diverticula, vascular malformations, and iatrogenic or traumatic injuries [2,3,4,5,6].

**Figure 1:**
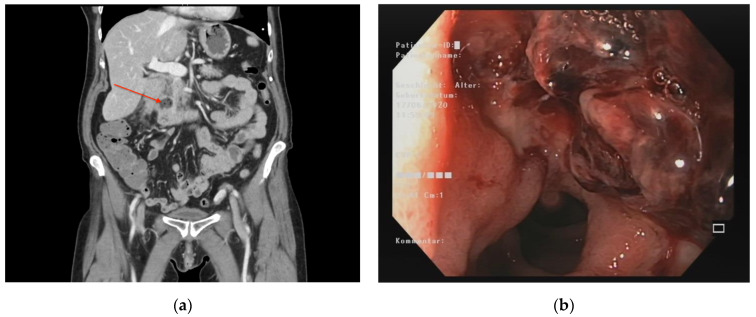
(**a**) CT scan. In the coronal reconstruction of the portovenous contrast-enhanced phase detection on a perforated duodenal diverticulum (red arrow) as a cause of peritonitis and sepsis. (**b**) Endoscopy. Chronic peptic duodenal ulcer with penetration into the hepatopancreatic duct.

The treatment of simple pancreaticobiliary disorders in early disease stages is a standardized procedure with different therapeutic strategies (symptomatic, interventional, and operative) for gastroenterologists, interventional radiologists, and visceral surgeons and is well described in multiple studies [7,8,9]. If standardized therapy fails, a bleeding or free organ perforation with severe complications occurs, therapeutic options are limited, and an individual approach is necessary. The rare case of severe complications such as acute uncontrollable bleeding, penetration into surrounding tissue, or sepsis is a therapeutic challenge (Figure 1). Particularly complex duodenal ulcers with penetration into surrounding tissue such as the hepatopancreatic ampulla, the common pancreaticobiliary channel, the pancreatic duct, the bile duct, branches of the gastroduodenal artery, the inferior pancreaticoduodenal artery, or the pancreatic head as well as duodenal scarring with duodenal and pyloric stenosis are leading to severe complications [10]. Due to the complex anatomy and acute bleeding or duodenal and pyloric stenosis, therapeutic endoscopic options are very limited. If less invasive procedures are exhausted, an emergency pancreaticoduodenectomy (EPD) can be the only option left [11].

Pancreaticoduodenectomy (PD) is one of the most challenging surgical procedures, which requires a high level of surgical expertise and is mainly performed for malignancy of the pancreatic head [12]. Performed in high-volume centers, in recent decades, the mortality rate has dropped from more than 20% to less than 5% for elective cases [13]. Although significant progress in reducing mortality has been achieved, even in elective operations, PD remains one of the most complicated surgical procedures in visceral surgery [14].

In contrast to elective PD, patients undergoing PD in an emergency setting are not well prepared and selected or suffered from extensive blood loss prior to surgery. While multiple studies have analyzed the outcome of elective PD, the topic of EPD has been scarcely investigated. Only up to 2% of PDs are performed for emergency indications [11,15,16]. Indications leading to EPD vary greatly from penetrating or blunt abdominal trauma to uncontrollable bleeding from ulcers, tumors, ruptured aneurysms, bleeding pseudocysts, duodenal perforation or diverticula, and iatrogenic causes [17]. The available literature mainly includes case reports or small patient series. However, considerable morbidity and mortality rates have been reported [11,17].

The aim of this study was to analyze a single-center experience of EPD for benign non-trauma indications and to review the literature concerning EPD.

## 2. Materials and Methods

Data on all patients undergoing PD at our institution between January 2015 and January 2022 were analyzed regarding indications leading to PD. Only cases of EPD for benign non-trauma indications were included. EPD has been defined as PD in an emergency setting, performed less than 12 h after the first diagnosis leading to indication. Cases with EPD performed for abdominal trauma, malignancies, and iatrogenic injury were excluded. Other exclusion criteria were PD in revision surgery and technical approaches different than the classic Whipple’s procedure with partial PD and reconstruction through pancreaticojejunostomy, gastrojejunostomy, and hepaticojejunostomy.

All cases of EPD meeting the inclusion criteria were analyzed. Data were analyzed regarding sex, age, indication, operative parameters (operation time, transfusion requirement), postoperative morbidity and mortality, length of ICU stay, and total hospital stay.

**Figure 2:**
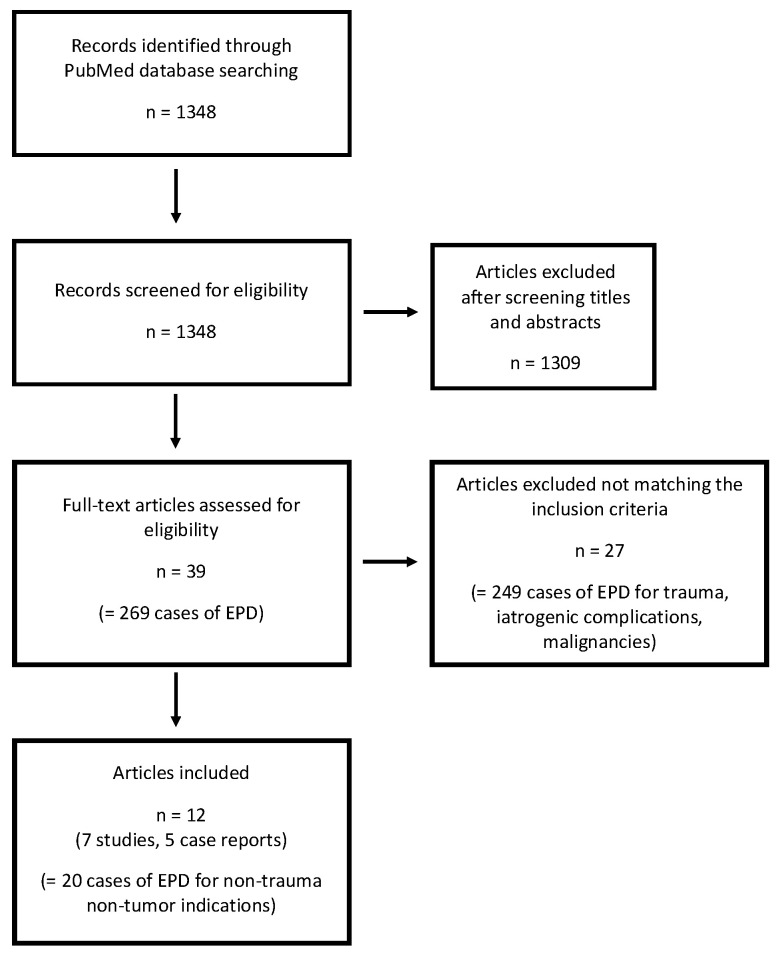
Flow diagram of the search strategy for reported cases of EPD.

Furthermore, we performed a structured keyword search using the PubMed database and reviewed reported cases of EPD. PubMed was searched for articles published between 1990 and 2022 using the keywords (“pancreaticoduodenectomy” OR “pancreatoduodenectomy” OR “Whipple procedure” OR “pancreatectomy” OR “pancreatic resection” OR “pancreatic surgery”) AND (“emergency” OR “emergent” OR “urgent” OR “acute” OR “non-trauma” OR “trauma” OR “perforation” OR “ulceration” OR “bleeding” OR “tumor”). Publications in languages other than English were excluded from the analysis. Two readers reviewed the title and abstract of selected publications to verify the inclusion and the full text of the selected articles. All recorded cases of classic Whipple’s procedure with primary reconstruction performed in an emergency setting for benign non-trauma indications were analyzed regarding indication and mortality. Detailed information of the search strategy for reported cases of EPD is given in Figure 2.

## 3. Results

Between January 2015 and January 2022, a total of 196 PDs were performed in our institution. The main indications leading to PD were adenocarcinoma of the pancreatic head (67 cases, 34.1%), chronic pancreatitis (28 cases, 14.4%), and others such as intraductal papillary mucinous neoplasm (IPMN), periampullary cancer, and neuroendocrine tumors (NET) of the pancreas (101 cases, 51.5%).

A total of 11 cases of EPD (5.6%) performed for benign non-trauma indications were identified (5 female and 6 male, mean age 63.7 years (SD 8.5 years, range 78–51)). Indications were duodenal ulcer with acute uncontrollable bleeding and penetration into the pancreas (6 patients, 54.5%), peptic duodenal ulcer with penetration into the hepatopancreatic duct and the pancreas (4 patients, 36.4%), and a massive perforated duodenal diverticulum with peritonitis and sepsis (1 patient, 9.1%). The patient’s clinical symptoms were signs of an acute abdomen with abdominal pain (54.5%), hemorrhagic shock (18.2%), or sepsis (36.4%). Preoperative diagnostics included blood sampling, contrast-enhanced multi-detector-computed tomography of the abdomen (GE Revolution HR, USA), and, in some cases, esophagogastroduodenoscopy. The surgical procedure was a non-pylorus-preserving partial pancreaticoduodenectomy (classic Whipple procedure) with pancreaticojejunostomy, gastrojejunostomy, and hepaticojejunostomy (11 Patients, 100%). The mean operative time was 369 min (SD 64.2 min, range 470–279). Perioperative transfusion of red blood cell concentrates was required in five cases (45.4%). The mean postoperative ICU stay was 8.6 days (SD 9,4 days, range 36–3) with a mean length of total hospital stay of 35.8 days (range 95–12 days). Postoperative complications (Clavien IIIa/b and V) occurred in 4 out of 11 patients (36.4%). In one patient (Clavien IIIb = 9.1%), operative revision of the biliodigestive anastomosis (BDA) because of an anastomotic leak was necessary; in two cases (Clavien IIIa = 18.0%), a temporary 8.5F percutaneous transhepatic cholangio-drainage (PTCD) placement was needed to gain healing of the BDA. One patient (Clavien V = 9.1%) developed acute necrotizing pancreatitis of the pancreatic remnant and colonic ischemia. This patient died after operative revision. Total 90-day postoperative mortality was 9.1% (1 patient). Detailed patient data are given in Table 1.

We reviewed 17 studies and 22 case reports, producing a total of 269 cases of EPD reported in the literature. The main indications were blunt or penetrating abdominal trauma (72.9%), iatrogenic complications (10.0%), bleeding malignancies (8.9%), and others (8.2%). Only 20 cases of EPD performed for benign non-trauma indications are reported in the literature. Indications were duodenal bleeding (10 patients, 50.0%), duodenal ulcer (4 patients, 20%), perforated duodenal diverticulum (3 patients, 15%), duodenal necrosis (1 patient, 5%, arteriocholedochal fistula (1 patient, 5%), and bile duct necrosis (1 patient, 5%). Postoperative mortality was 35% (7/20 patients).

Detailed patient data are given in Table 2.

Combined with our 11 cases, 31 cases of EPD for benign non-trauma indications are reported worldwide. The main indications are acute duodenal bleeding (ulcer, vascular malformation, pseudocyst) (51.5%), duodenal ulcer (24.2%), and perforated duodenal diverticula (12.1%). Postoperative mortality was 25.8% (8/31 patients).

## 4. Discussion

In the last decade, centralization of pancreatic surgery, improvement of surgical techniques, and perioperative management have decreased surgical morbidity and mortality rates of elective PD [13,27,28]. Although PD is an established procedure in elective surgery, PD in an emergency setting still remains a rare event. Studies analyzing the indications for PD have shown that only up to 2% of PDs are performed in an emergency setting [11,15,16]. Indications leading to EPD have been scarcely investigated and analyzed. Our literature review has shown that the main indications for EPD are blunt or penetrating abdominal trauma caused by motor vehicle accidents, gunshot wounds, and stab wounds. Other indications, such as i.e., bleeding from malignancies and others, describe a very inhomogeneous group of etiologies. Assuming that up to 2% of PDs are performed as EPDs, only 0.16% of PDs are performed for benign non-trauma indications in an emergency setting. Only small patient series and case reports are published in the literature. As part of our literature review, a total of 20 cases of EPD met our inclusion criteria.

Our series of 11 cases of EPD for benign non-trauma indications reflects one of the largest cohorts reported in the literature. Including our 11 cases, a total number of 31 cases of EPD for benign non-trauma indications are reported worldwide. The main indications are acute duodenal bleeding (ulcer, vascular malformation, pseudocyst), chronic duodenal ulcer with penetration into surrounding tissue and stenosis, and perforated duodenal diverticula.

There is a lack of studies analyzing the indication and outcome of patients undergoing EPD for benign non-trauma indications. Because of the different pathophysiologies leading to EPD, cases of abdominal trauma or iatrogenic cause are not comparable with non-trauma indications. Patients undergoing EPD for blunt or penetrating abdominal injury are younger and have fewer comorbidities [29,30]. Nevertheless, high mortality rates of up to 33.0% are described for trauma patients [29]. Morbidity rates vary from 51–100% [29,31]. In contrast, the long-term outcome seems favorable in patients with EPD for abdominal trauma or iatrogenic cause [31,32]. Patients with bleeding malignancies are another large group regarding indications for EPD. The reported cases vary greatly from diffuse B-cell lymphoma and duodenal gastrointestinal stromal tumor (GIST) to bleeding metastasis from renal cell carcinoma and acute bleeding from ampullary adenocarcinoma [5,21,33,34,35]. Due to the wide range of bleeding malignancies and the different etiologies, these cases are not eligible to evaluate the outcome of EPD in non-trauma patients and should, therefore, be considered separately.

In addition to the already rare literature with small patient numbers, available reports combine the different indications leading to EPD, which reduces the validity of the results.

Our results show that EPD for benign non-trauma indications can be performed with acceptable morbidity rates. Morbidity rates of 36.4% in EPDs are comparable to morbidity rates in PDs performed in an elective setting, varying from 25.9% to 48.6% [36,37,38]. The most relevant post-surgical complications are anastomotic leak of the BDA and pancreatic fistula. The management is identical in both PD and EPD. In centers with a high level of experience in pancreatic surgery, postoperative complications after pancreatectomy can be treated with good results [37]. A decisive factor for successful complication management is an interdisciplinary collaboration between surgeons, interventional radiologists, and gastroenterologists.

In our case series with 11 patients, postoperative mortality was only 9.1% (1 patient). The data analysis of all available cases of EPD for benign non-trauma indications showed a higher postoperative mortality rate of 25.8% (8/31 patients). Although operations were performed for emergency indications with acute bleeding and unstable patients, there was no case of intraoperative mortality. Patient death after EPD occurs exclusively due to postoperative complications. The main postoperative complications leading to death are systemic organ complications with multiorgan failure. Individual comorbidity and risk factors increase postoperative mortality in EPD.

The essential prerequisite for successful therapy with EPD is the preoperative selection of eligible patients. While patients in elective PDs are well selected and prepared, preoperative diagnostics in patients undergoing EPD are possible to a lesser extent. If less invasive approaches such as angiographic occlusion or endoscopic clipping are exhausted, the indication for EPD is an individual decision of the attending surgeon together with the relevant departments. Only surgeons with a high level of experience in hepatobiliary and pancreatic surgery should make decisions leading to EPD.

## 5. Conclusions

In conclusion, EPD for benign non-trauma indications is a rare event, mainly performed for acute duodenal bleeding, perforated, and/or bleeding duodenal ulcer, and perforated duodenal diverticula. Regarding data on all reported cases of EPD for benign non-trauma indications, increased postoperative mortality rates are reported (25.8%). If less invasive approaches are exhausted, EPD is a life-saving procedure with acceptable results in strictly selected patients. Performed by surgeons with a high level of expertise in hepatobiliary and pancreatic surgery, surgical morbidity and mortality rates in EPD can be decreased.

## Figures and Tables

**Table 1:** Details of patients undergoing emergency pancreaticoduodenectomy (EPD) at our institution.

Patient No.	Age/Gender	Indication	Procedure	Complication	Hospital Stay (Days)	Death
1	70/F	perforated duodenal diverticulum	cl Whipple	no	14	no
2	65/F	bleeding ulcer	cl Whipple	BDA insufficiency	95	no
3	66/F	complex duodenal ulcer	cl Whipple	no	23	no
4	64/M	complex duodenal ulcer	cl Whipple	no	21	no
5	51/M	bleeding ulcer	cl Whipple	no	20	no
6	72/F	complex duodenal ulcer	cl Whipple	no	21	no
7	55/M	bleeding ulcer	cl Whipple	BDA insufficiency	45	no
8	78/M	bleeding ulcer	cl Whipple	BDA insufficiency	44	no
9	51/M	complex duodenal ulcer	cl Whipple	no	63	no
10	62/M	bleeding ulcer	cl Whipple	no	12	no
11	67/F	bleeding ulcer	cl Whipple	Acute necrotizing pancreatitis, colonic ischemia	(8) *	yes

cl Whipple = classical Whipple procedure; BDA = biliodigestive anastomosis; * death after 8 days postoperative.

**Table 2:** Cases of emergency pancreaticoduodenectomy (EPD) performed for benign non-trauma indications reported in the literature.

Study Year	Author	Patients Meeting Inclusion Criteria	Indication	Mortality Rate
2002	Z´graggen et al. [18]	1/4	duodenal ulcer	0/1, 0%
2013	Gulla et al. [19]	1/10	bleeding duodenal ulcer	0/1, 0%
2015	Strobel et al. [11]	2/10	bleeding duodenal ulcer, bile duct necrosis	2/2, 100%
2016	Nentwich et al. [16]	4/10	duodenal ulcer, bleeding pancreatic pseudocyst, perforated duodenal diverticulum	2/4, 50%
2017	Eftimie et al. [20]	1/1	bleeding pseudoaneurysm	0/1, 0%
2017	Lupascu et al. [21]	3/5	bleeding pancreatic pseudocyst, bleeding duodenal diverticulum, duodenal varices	1/3, 33%
2017	Tsai et al. [22]	2/6	bleeding duodenal ulcer, bowel ischemia	1/2, 50%
2019	Ahmad et al. [23]	1/1	arteriocholedochal fistula	0/1, 0%
2019	Philip et al. [24]	1/1	perforated duodenal diverticulum	0/1, 0%
2021	Kitazono et al. [25]	1/1	bleeding arteriovenous malformation	0/1, 0%
2021	Tian et al. [6]	1/1	duodenal diverticulum	0/1, 0%
2022	Schlanger et al. [26]	2/4	bleeding pseudoaneurysm	1/2, 50%

## Data Availability

The data published in this research are available on reasonable request from the first author and corresponding authors.

## Abbreviations

EPD	Emergency Pancreaticoduodenectomy
PD	Pancreaticoduodenectomy
ICU	Intensive Care Unit
NET	Neuroendocrine Tumor
IPMN	Intraductal Papillary Mucinous Neoplasm
BDA	Biliodigestive Anastomosis
PTCD	Percutaneous Transhepatic Cholangiography and Drainage
GIST	Gastrointestinal Stromal Tumor
